# A Light Scattering Investigation of Enzymatic Gelation in Self-Assembling Peptides

**DOI:** 10.3390/gels9040347

**Published:** 2023-04-19

**Authors:** Stefano Buzzaccaro, Vincenzo Ruzzi, Fabrizio Gelain, Roberto Piazza

**Affiliations:** 1 Department of Chemistry, Materials Science, and Chemical Engineering (CMIC), Politecnico di Milano, Edificio 6, Piazza Leonardo da Vinci 32, 20133 Milano, Italy; 2 Unità di Ingegneria Tissutale, Fondazione IRCCS Casa Sollievo della Sofferenza, 71013 San Giovanni Rotondo, Italy; 3 Center for Nanomedicine and Tissue Engineering, ASST GOM Niguarda, 20162 Milano, Italy

**Keywords:** gels, self-assembling peptide, photon correlation imaging, DLS, enzymatic gelation

## Abstract

Self-assembling peptides (SAPs) have been increasingly studied as hydrogel–former gelators because they can create biocompatible environments. A common strategy to trigger gelation, is to use a pH variation, but most methods result in a change in pH that is too rapid, leading to gels with hardly reproducible properties. Here, we use the urea–urease reaction to tune gel properties, by a slow and uniform pH increase. We were able to produce very homogeneous and transparent gels at several SAP concentrations, ranging from c=1g/L to c=10g/L. In addition, by exploiting such a pH control strategy, and combining photon correlation imaging with dynamic light scattering measurements, we managed to unravel the mechanism by which gelation occurs in solutions of ${\left(\mathrm{LDLK}\right)}_{3}$-based SAPs. We found that, in diluted and concentrated solutions, gelation follows different pathways. This leads to gels with different microscopic dynamics and capability of trapping nanoparticles. At high concentrations, a strong gel is formed, made of relatively thick and rigid branches that firmly entrap nanoparticles. By contrast, the gel formed in dilute conditions is weaker, characterized by entanglements and crosslinks of very thin and flexible filaments. The gel is still able to entrap nanoparticles, but their motion is not completely arrested. These different gel morphologies can potentially be exploited for controlled multiple drug release.

## 1. Introduction

The spontaneous assembly of a wide variety of molecules, called gelators, dispersed in water, under the influence of different non-covalent forces such as hydrogen bonding, π-stacking, hydrophobic, and ionic forces [1,2,3,4], can lead to the formation of molecular hydrogels. These materials have sparked great interest for their possible use in controlled drug release [5,6,7,8], regenerative medicine [9], and tissue engineering [10,11,12].

In this context, the key strategy is to develop synthetic biomimetic hydrogels, that reproduce many of the properties of the extracellular matrix (ECM) [13,14,15]. In recent years, self-assembling peptides (SAPs) have been commonly used as gelators, because they form nanofibrous matrices that resemble the fibrillar matrix of the natural ECM [16]. In order to trigger the self-assembly, an external stimulus is usually needed [4,17], such as adding a co-solvent [18,19,20,21], irradiating the sample with UV light [22,23], varying the temperature [24,25], or changing the ionic strength [26] or the pH of the system [27,28,29,30,31].

In the case of SAPs that respond to a pH change, the gelators contain a pH-sensitive functionality. The degree of ionization of the functional group governs the solubility of these molecules. The gelators can be classified as acid-triggered or base-triggered [2], depending on the properties of the ionizable group. In early attempts, the pH was varied by adding a strong acid or base to the peptide liquid solution [30,31]. However, this led to a rapid change in pH, leading to heterogeneous gels, with properties that were difficult to replicate, because the rate of mixing of the components was slower than the rate of gelation [27,32]. To overcome this issue, it was suggested to use an additive that slowly hydrolyses with water, forming an acid or a base. If the dissolution of the additive is much faster than the hydrolysis, a homogeneous medium is generated, and a uniform pH variation throughout the system takes place. Because a larger number of SAPs can form gels when a solution at high pH is acidified, the first method that exploited this strategy used glucono-δ-lactone (GdL) [32]. The hydrolysis of GdL is slow and gives gluconic acid, which decreases the pH in a controllable way [33]. The use of a kinetically controlled acidification, resulted in reproducible and uniform gels, and the improvement in gel homogeneity translated into improved mechanical properties [32]. Only recently, a similar strategy was employed to control the gelation of base-triggered SAPs. In this case, a controlled alkalinization of the solution was achieved by using the urea–urease reaction [28], that involves hydrolysis of urea by urease and production of ammonia, increasing the pH [2]. While the concentration of GdL controls both the final gel pH and the rate of the gelation process, the use of the urea–urease reaction, allows these two aspects to be independently controlled. In fact, the hydrolysis rate depends on the concentration of urease, whereas the amount of urea sets the final pH. A further benefit of the use of enzyme-induced gelation, is that the rate of hydrolysis is sufficiently slow that observation of the SAP assembly process is possible [32,34].

While the micro-structure of peptide-based hydrogels, and their mechanical properties, have been extensively studied, with several techniques [21,35,36], such as rheometry [37,38,39], static small-angle scattering [40,41], and transmission electron microscopy [42,43], the kinetics of aggregation and the following gelation have been investigated much less. A relatively small number of works have investigated the assembly process using circular dichroism (CD) [44,45,46,47], Fourier-transform infrared spectroscopy (FTIR) [48], and thioflavin T fluorescence [34,49]. To our knowledge, light scattering studies of the SAP self-assembly kinetics, are still lacking. The main aim of this work, is to show that a fruitful combination of enzyme-controlled pH variation with photon correlation imaging (PCI) and dynamic light scattering (DLS) measurements, can help to elucidate some aspects of the mechanism that governs the gel formation in SAP solutions.

## 2. Results and Discussion

### 2.1. pH Evolution in the Urea–Urease Reaction: Optimizing Parameters to Obtain Homogeneous Gels

**Figure 1:**
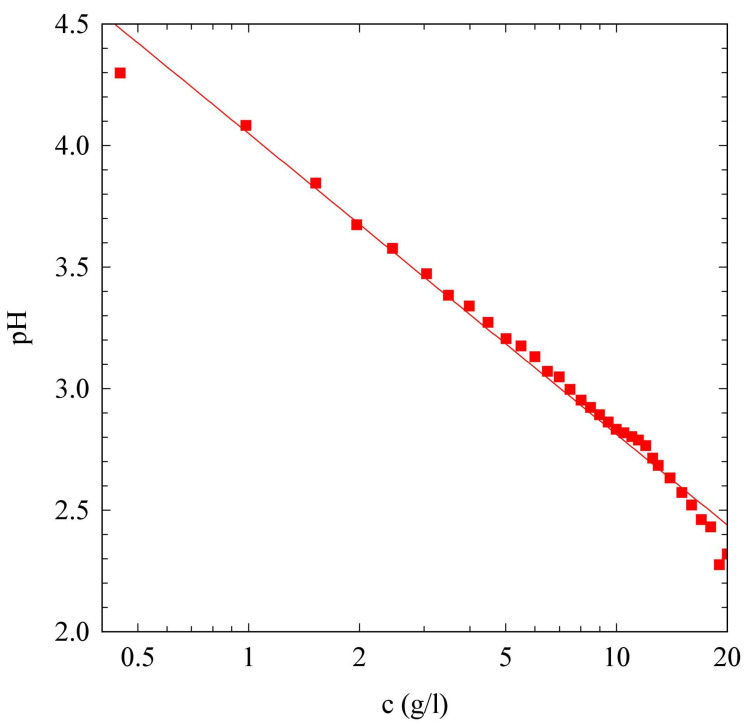
Dependence of the solution pH on the SAP concentration (log scale). The continuous line is a logarithmic fit, $\mathrm{pH}=-1.24\log(5.36\times 10^{-4}c)$.

When dissolved in water, SAPs show an acidic behavior, characterized by a logarithmic decay of the solution pH with the peptide concentration (see Figure 1). This acidic behavior of the SAPs, is important to define the amount of acid to add to the solution in order to slow down the hydrolysis kinetics. To obtain a homogeneous gel, it is important that the peptide dissolution is significantly faster than the urea hydrolysis. This leads to a uniform pH variation throughout the system. It is well known that the activity of urease strongly depends on pH, with a maximum rate of conversion around pH=7 [50,51]. For our purpose, it is important to work with a low initial pH of the solution, in order to reduce the activity of the enzyme. When the initial pH is below 4, the production of ammonia is limited. However, after a certain time, a rapid conversion to the high pH state occurs [52]. In these conditions, the urea–urease reaction is characterized by a lag-phase, whose duration can be controlled by the nature and concentration of the acid used [2]. We found that adding 0.012mM of acetic acid to our solution, reduced the initial pH value to a value of 3.7, that is optimal for our purpose.

**Figure 2:**
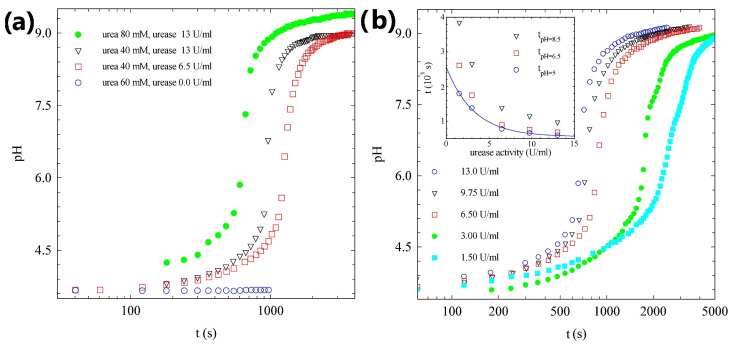
(**a**) Time evolution of the pH in the urea–urease reaction, for samples characterized by the same amount of acetic acid (0.012mM) but different concentrations of urea and urease, as indicated in the legend. (**b**) Time evolution of the pH in the urea–urease reaction, for samples characterized by the same amount of acetic acid (0.012mM) and urea (60mM), but different urease activity. Inset: time needed to reach pH=5 (circles), pH=6.5 (squares), and pH=8.5 (triangles). The continuous line is an exponential fit.

Panel (a) of Figure 2, shows the urea–urease pH kinetics at different values of urea and urease, at a fixed acetic acid concentration. In the absence of the enzyme, as expected, the pH of the solution does not change. When we add urease, the data show that the rate of alkalinization is slow below pH = 5. In fact, ammonia salts are formed and the acid is neutralized. Using acetic acid, a weak acid, we produce acetic acid–ammonium acetate, a buffer that resists the pH change [50]. The time evolution of the reaction is governed by the initial concentrations of urea and urease. Only the amount of urea sets the final pH: the larger the amount of urea, the higher the final pH. In strongly acidic conditions, the formation of ammonia–ammonium buffer limits the maximum pH that can be reached [2,50,53]. On the contrary, the value of the lag-time is related both to the amount of urea and urease [28,50]. If we compare the two samples with the same amount of urea (40mM, squares and triangles in Figure 2), we note that the lag-time changes from 1000s to 1300s, on halving the enzyme concentration. If we focus on the curves with the same urease concentration (13U/mL, triangles and full dots in Figure 2), we note that when doubling the amount of urea, the lag-time shows a thirty percent reduction. In order to obtain final gels with the same pH, we decided to work with a fixed concentration of urea, equal to 60mM, and tune the lag-time, simply by changing the amount of enzyme we used.

Panel (b) of Figure 2, shows the urea–urease pH kinetics for different values of urease concentration. As expected, the initial values of pH are the same for all the samples, fixed by the acetic acid concentration. In addition, the final pHs are the same, and they are set by the amount of urea in solution. The lag-phase increases for lower concentrations of urease, because the production of ammonia is slower. We note that all the curves show a slow pH rate increase for pH<5, due to the acid–ammonium acetate buffer effect, a rapid increase in the rate between 5<pH<8.5, and a pronounced slowing down of the pH growth rate when the ammonia–ammonium buffer concentration is no longer negligible. To be more quantitative, in the inset of Figure 2b, we plot, for each urease concentration we studied, the times at which the samples reach pH values equal to 5, 6.5, and 8.5. In the limited range of urease concentrations we studied, the lag-time, arbitrarily defined as the time at which the sample reaches pH=5, decreases exponentially with the urease activity, and seems to reach an asymptotic value of about 9 min. Our data also clearly indicate that the duration of the phase of rapid increase in pH, is strongly dependent on the urease concentration. In fact, for a urease activity equal to 13U/mL, the sample takes around 5min to change from pH=5 to pH=8.5, whereas for the minimum urease concentration we studied, the system needs more than 30min to show the same pH increase. In the following section, we focus on samples prepared with a urease concentration of 13U/mL.

**Figure 3:**
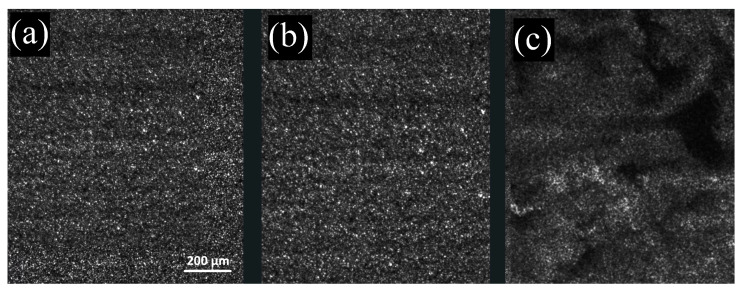
(**a**) Speckle pattern of sample H, two hours after its preparation. (**b**) Speckle pattern of sample L, two hours after its preparation. (**c**) Speckle pattern of a sample with the same composition as sample L, but without the addition of acetic acid, two hours after its preparation. In this case, a heterogeneous speckle pattern is clearly visible, demonstrating the sample’s inhomogeneity.

The preliminary study of the hydrolysis reaction kinetics in the presence of acetic acid, allowed us to find the best conditions to obtain homogeneous SAP gels. In fact, while the addition of NaOH resulted in an instantaneous pH jump, inducing a gelation that begins just after addition of the base, yielding a non-homogeneous and whitish gel, exploiting the urea–urease reaction to change the pH of the solution, allows us to obtain very transparent and homogeneous gels (panels (a) and (b) of Figure 3) at several SAP concentrations, ranging from c=1g/L to c=10g/L. At higher SAP concentrations, and a fixed acetic acid dilution, the gelation kinetics were not reliable. This is probably due to an initial pH value that was too low to initiate the urea hydrolysis. In fact, urease loses activity at pH≃3 or below [2]. To obtain consistent results, it is important to keep the initial pH between 3.5 and 4. To emphasize the importance of the slow variation in the pH, in panel (c) of Figure 3, we show the speckle pattern measured for a gel obtained with the same urea, urease, and SAP concentrations as the sample in panel (b), but without the addition of the acetic acid. In this case, the initial pH is not sufficiently low, and the urea–urease reaction is so fast that the sample starts gelating during the mixing. The final gel, as expected, is not homogeneous, and keeps memory of the mixing dynamics.

In what follows, we will focus on two samples, characterized by the two SAP concentrations that bounded the interval we investigated: the sample at c=10g/L will be referred to as Sample H and the less diluted sample, prepared at c=1g/L, will be referred to as Sample L.

### 2.2. Light Scattering Study of the SAPs’ Aggregation Kinetics

#### 2.2.1. Sample H

**Figure 4:**
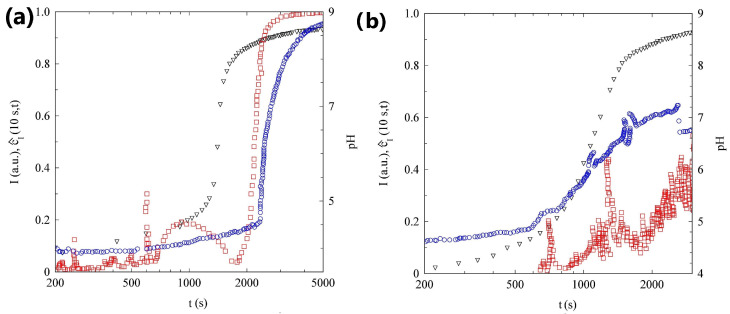
(**a**) Temporal evolution of pH (triangles), scattered intensity (circles), and ${\hat{c}}_{I}\left(10\;s,t\right)$ (squares) of sample H. (**b**) Temporal evolution of pH (triangles), scattered intensity (circles), and ${\hat{c}}_{I}\left(10\;s,t\right)$ (squares) of sample L.

In panel (a) of Figure 4, we plot the evolution with time of pH, scattered intensity, and normalized degree of the time correlation ${\hat{c}}_{I}\left(\tau =10\;s,t\right)$. After 2400s, the data show a marked growth in the scattering intensity. Eventually the intensity reaches a value about 15 times larger than its initial value. This intensity increase takes places about 1000s after the end of the pH lag-time. Interestingly, a small but continuous growth in the scattering intensity can also be observed during the pH lag-time. The variation in scattered light is due to a change in the sample structure factor, suggesting that the SAPs also start to slightly aggregate during the pH lag-time. It is therefore tempting to state, that the gel formation coincides with the abrupt increase in the scattering intensity.

However, the data in Figure 4a suggest a slightly different scenario. Before the sample gels, because the decay time of ICS for a fluid SAP is at least three orders of magnitude faster than 10s, the correlation index is close to zero and quite noisy. However, around $t_{g}=1800\;s$, ${\hat{c}}_{I}\left(\tau =10\;s,t\right)$ shows a rapid increase, reaching a value of about 1 in a few minutes. A value close to 1, indicates that the speckle pattern is fully arrested on the time scale of 10s. The whole sample turned quite rapidly into an arrested gel. It is important to stress that the sample gelation *anticipates* the rapid intensity growth, by about 10min.

**Figure 5:**
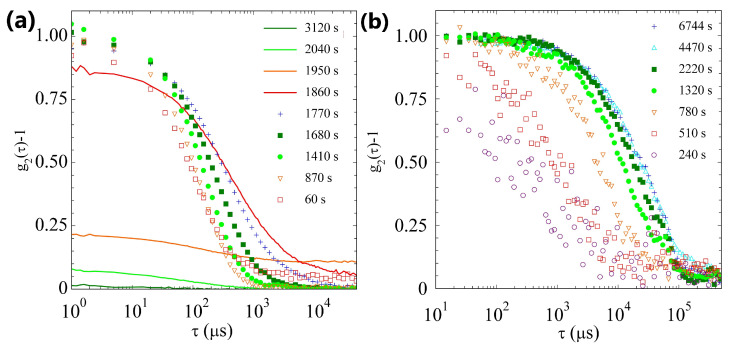
(**a**) ICFs at different times, indicated in the legend, for sample H. Symbols stand for ergodic samples, while continuous lines indicate ICFs taken after $t_{g}$, when the time averaged ICF is no longer reliable. (**b**) ICFs at different times, indicated in the legend, for sample L.

Unfortunately, the PCI temporal resolution does not allow us to monitor the sample dynamics during the pH lag-time phase and in the first phases of the gelation process. To overcome this limitation, we exploit the capability of DLS to monitor the fast dynamics of non-arrested systems. In panel (a) of Figure 5, we plot the ICFs measured at different times, starting 60s after the sample preparation. Our data indicate that, before $t_{g}$, the ICFs have a contrast close to one and fully decorrelate, thus the sample is still liquid. In this phase the sample dynamics slow down, suggesting the formation of larger aggregates, in accordance with the slow growth in the scattering intensity, monitored by PCI. The contrast of the intensity correlation function decreases abruptly and goes to zero for $t>t_{g}$, suggesting that a non-ergodic arrested gel phase is formed.

#### 2.2.2. Sample L

In panel (b) of Figure 4, we plot the evolution with time of the pH, scattered intensity, and of the normalized degree of the time correlation ${\hat{c}}_{I}\left(\tau =10\;s,t\right)$, for sample L. The scattering intensity of this sample slowly increases with time, up to a factor of about 5. The data are rather noisy, because the sample concentration is an order of magnitude smaller than in the previous case: the light scattered by this sample is close to the detection limit of our PCI setup. The intensity growth rate still increases at the end of the pH lag-phase, but the rise is much milder than in the more concentrated sample. In addition, the variation in the correlation index is much more limited and the system is never fully arrested for τ=10s. Removing the cell from the PCI setup and turning it upside down, suggests that a very viscous solution, possibly a gel with a very low yield stress, forms.

In this case too, we resort to DLS to better characterize the sample’s dynamics. Panel (b) of Figure 5, shows the ICFs measured at several times, starting 240s after the sample preparation. Initially, the scattered light is low and the correlation function is very noisy. The scattered intensity increases with time and the correlation functions are more reliable. Our data indicate a continuous slowing down of the dynamics, but without a detectable decrease in the contrast. The sample, at least at the q-vector we are probing, remains ergodic and never shows arrested dynamics.

#### 2.2.3. Comparison of Samples’ Dynamics

**Figure 6:**
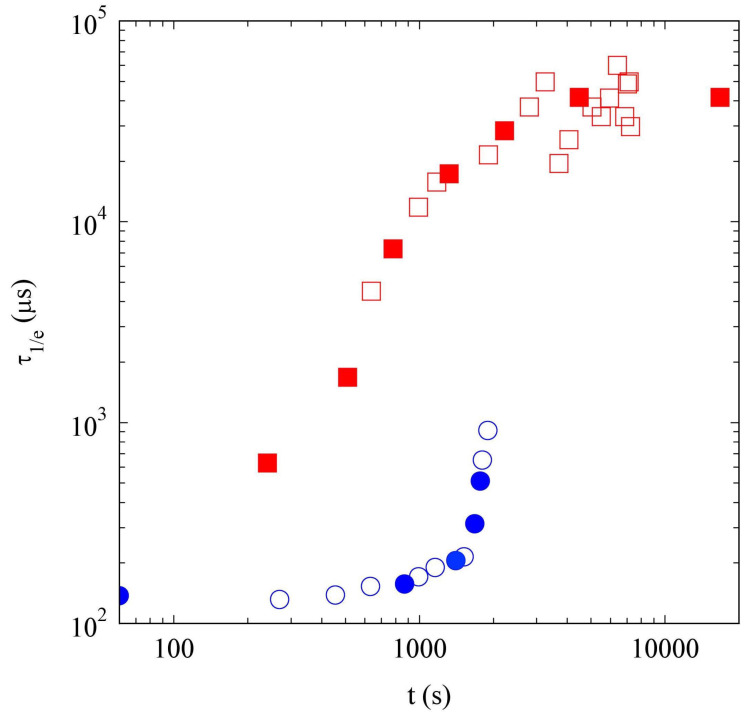
Decay times of the ICFs $\tau_{1/e}$ as a function of time, for samples H (circles) and L (squares). Full points refer to the ICFs shown in Figure 5.

We report in Figure 6, the decay time of the ICFs, $\tau_{1/e}$, defined as $g_{2}\left(\tau_{1/e}\right)-1=e^{-1}$, measured for both samples H and L, as a function of time. The two samples, as already discussed, show a radically different behavior. For sample H, the decay time initially grows slowly, until it rapidly diverges at the gelation time. On the contrary, for the L sample, the value of $\tau_{1/e}$ increases with time, reaching a large but *finite* value, almost two orders of magnitude larger than the initial one. We note, quite unexpectedly, that when both the samples are still liquid, the dynamics of sample L are, at least, an order of magnitude slower than the dynamics of sample H. This suggests that the aggregates formed in the dilute solution are less dense but occupy a larger volume.

### 2.3. Light Scattering Study of the SAPs Solution Seeded with Nanoparticles

The above results do not completely rule out the formation of an arrested phase for sample L. In fact, in principle, its dynamics could be arrested at lower q-vectors. Unfortunately, DLS experiments at low angles, where the dynamics are slower, in samples whose dynamics are not stationary in time, is a very challenging task, mainly because it is difficult to ensure that the duration, δt, of a DLS experiment is still much shorter than the characteristic timescale of the sample evolution. For this reason we decided to adopt a different and complementary approach. We seeded our sample with small colloidal particles, with a diameter of σ=192nm. If SAPs form an arrested phase, we would expect that the motion of the tracers would be limited, if not fully arrested.

#### 2.3.1. Sample H

In the case of seeded samples, the scattered light mainly originates from the tracer particles. In our experiments we do not observe any signs of intensity variation, suggesting that the SAP aggregation does not promote particle clustering.

**Figure 7:**
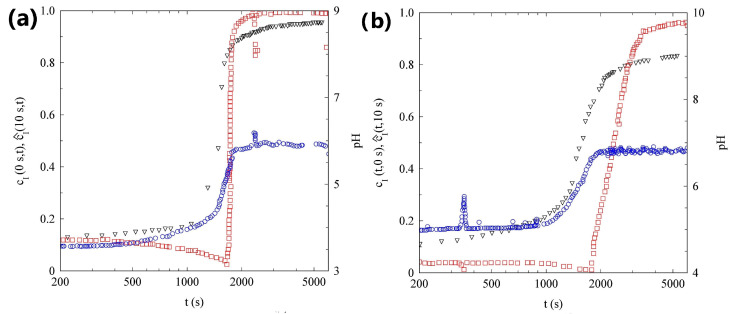
(**a**) Temporal evolution of the pH (triangles), $c_{I}\left(0\;s,t\right)$ (circles), and ${\hat{c}}_{I}\left(10\;s,t\right)$ (squares) of sample H seeded with PMMA nanoparticles. (**b**) Temporal evolution of the pH (triangles), $c_{I}\left(0\;s,t\right)$ (circles), and ${\hat{c}}_{I}\left(10\;s,t\right)$ (squares) of sample L seeded with PMMA nanoparticles.

In panel (a) of Figure 7, we plot the temporal evolution of pH, $c_{I}\left(\tau =0\;s,t\right)$, and ${\hat{c}}_{I}\left(\tau =10\;s,t\right)$ for the more concentrated sample. We note that $c_{I}\left(\tau =0\;s,t\right)$, slightly increasing during the initial part of the experiment, shows a marked growth at t=1500s, about 500s after the end of the lag-phase, and levels off when the pH growth rate rapidly decreases.

Since our exposure time is $t_{e}=10\;\mathrm{ms}$, the marked increase in $c_{I}\left(\tau =0\;s,t\right)$ indicates that the characteristic timescale of the tracer dynamics is smaller than 10ms during the pH lag-phase, but rapidly increases approaching the gel phase. In fact, when $c_{I}\left(\tau =0\;s,t\right)$ reaches a stationary value, suggesting that the dynamics appear static on the timescale of $t_{e}$, the ${\hat{c}}_{I}\left(\tau =10\;s,t\right)$ starts to increase and in a few minutes reaches a value close to 1, indicating that the tracers are arrested inside the gel matrix.

**Figure 8:**
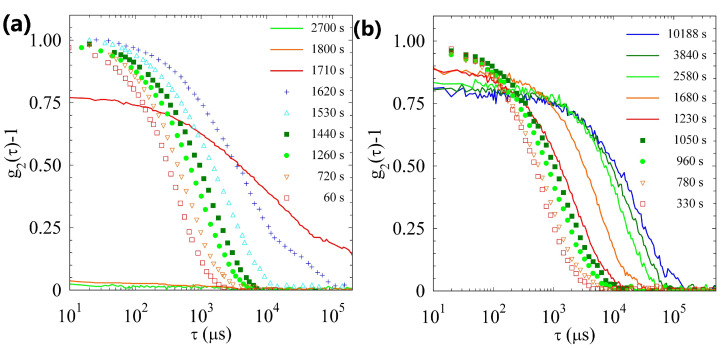
(**a**) ICFs at different times, indicated in the legend, for sample H seeded with PMMA nanoparticles. (**b**) ICFs at different times, indicated in the legend, for sample L seeded with PMMA nanoparticles. In both panels, symbols stand for ergodic samples, while continuous lines indicate ICFs taken after $t_{g}$, when the time averaged ICF is no longer reliable.

This picture is confirmed by the ICF measured by DLS, shown in panel (a) of Figure 8. The sample dynamics become slower with time but the ICF completely decays to zero in less of 10ms for t<1530s. This is consistent with the PCI analysis, that shows a growth in $c_{I}\left(\tau =0\;s,t\right)$ only for t>1500s. After t=1620s, the sample becomes non-ergodic and the contrast rapidly drops to zero in less than 3min. The sample is fully arrested for t>1710s, in accordance with the increase in $c_{I}\left(\tau =10\;s,t\right)$ starting at t=1700s.

#### 2.3.2. Sample L

The data in panel (b) of Figure 8, that refer to the most diluted sample, show the temporal evolution of pH, $c_{I}\left(\tau =0\;s,t\right)$, and ${\hat{c}}_{I}\left(\tau =10\;s,t\right)$. If compared with panel (a) of Figure 7, samples H and L show strong analogies: the $c_{I}\left(\tau =0\;s,t\right)$ increases at the end of the pH lag-phase and the tracer dynamics are fully arrested for t>4000s. The only qualitative difference concerns the speed of the gelation process: while for the concentrated sample the $c_{I}\left(\tau =10\;s,t\right)$ takes less than 2min to change from 0 to 1, in the case of the more diluted sample, about 20min are required.

Although the gelation of the two samples shares some similarities, the DLS measurements shown in panel (b) of Figure 8, allow us to observe a very important difference. The analysis of the ICFs, shows that the gelation process is characterized by three different stages: (I) For t<1050s, the sample is fluid and ergodic, the contrast is equal to 1, and the dynamics slows with time; (II) for t>1050s the contrast slowly decreases, reaching a final value around 0.77 for t=2580s. In this stage, the sample dynamics also slow down; (III) the contrast does not change anymore but the dynamics continue to slowly evolve. A final contrast of around 0.77, indicates that the motion of the tracers is not completely arrested at the probed length scale, the particles are, on average, bound to fixed positions, but they can still explore a region of the order of a few tens of nanometers. If we compare the sample evolution measured with DLS and PCI, we can see that the end of the first stage coincides with the time at which the $c_{I}\left(\tau =0\;s,t\right)$ starts to increase. The end of the second stage takes place when $c_{I}\left(\tau =10\;s,t\right)$ begins to level off.

### 2.4. Discussion

Our results show that the approach to a gel phase follows different pathways in diluted and concentrated SAP solutions. Figure 4 shows that there is a delay of a few minutes between the pH jump and the gel formation. Several studies of similar fibril–former gelators, showed the existence of a lag-phase, during which the peptide aggregation is limited, followed by a stage in which the aggregate concentration rapidly increases [54,55,56]. It was concluded that, in these SAP solutions, the self-assembly process is characterized by a nucleated reaction, and several mechanisms were proposed to explain such a process [57,58,59,60]. Panels (b) of Figure 4 and Figure 5, on the contrary, clearly indicate that, in a sufficiently diluted system, the raising of the pH *gives time* to the peptide aggregation. The difference becomes evident if we contrast the results of the DLS measurements, as shown in Figure 6. Sample L is characterized by a continuous slowing down of the dynamics, suggesting that quite large aggregates start to form from the beginning and their number and/or length increase with time. Conversely, in sample H, we note an initial very slow formation of smaller (and probably more compact) aggregates, as shown by the fact the decorrelation times are at least an order of magnitude lower than in the L sample. After a lag time of about 30min, there is a rapid increase in the aggregate size and number, that leads to gelation. Interestingly, this aggregation process continues after the gel formation, as demonstrated by the increase in the scattered light measured by PCI, for at least one hour.

The concurrence of different mechanisms of self-assembly in hydrogel-forming peptides, whose rates depend on the monomer concentration, has been observed previously [34]. In particular, the first mechanism proposed for fibril formation, is the aggregation of monomers to form stable clusters [61,62]. The association of new monomers to the ends of existing protofibrils, is responsible for the measured increase in the fibril mass concentration. This aggregation mechanism, usually called *first nucleation*, is an entirely monomer-dependent mechanism, and it is described as a homogeneous, uncatalyzed reaction [55]. This mechanism favors the formation of long and thin fibrils. A second proposed mechanism of new fibril formation, is a self-catalyzed process. The pre-existing fibrils act as nuclei for the fibrillar formation. This is a *secondary nucleation* mechanism: gelators nucleate on the surface of the existing aggregates [63]. This lateral growth favors the formation of more compact aggregates. Our results suggest that primary nucleation is the favored mechanism of self-assembly in the diluted system, while secondary nucleation becomes dominant when the peptide concentration increases.

The gels’ micro-structures and properties reflect these differences. At high concentration, a strong gel is formed, made of relatively thick and rigid branches that firmly entrap nanoparticles. On the scale of hundreds of nanometers probed by DLS, the system is completely arrested. In contrast, the gel formed in the diluted conditions is weaker, characterized by entanglements and crosslinks of very thin and flexible filaments. Between two crosslinked points, the motion of the strands is still possible and the dynamics is never arrested. The microscopic dynamics slows down with time, possibly reflecting the increase in filament stiffness as a consequence of the increase in diameter associated with monomers’ lateral adhesion, that take place after the gel formation. Moreover, the gel is able to entrap nanoparticles, but their motion is not completely arrested and reflects the non-arrested motion of the filamentous matrix.

## 3. Conclusions

In this work, we have used urea–urease hydrolysis to control the gelation of a base-triggered SAP system. The use of the urea–urease reaction, allows the final pH and the gelation rate to be independently controlled: the final pH is controlled by the amount of urea, whereas the hydrolysis rate depends on the concentration of urease. We were able to produce very homogeneous and transparent gels at several SAP concentrations, ranging from c=1g/L to c=10g/L. Better control on the gelation kinetic of SAPs, could be of crucial importance for several reasons: (1) it will improve the reproducibility of the scaffold mechanical properties; as a consequence (2) it will allow for a standardized reaction, related to the mechanical properties of a seeded cell in 3D cell culture systems, or scaffolds to be implanted; (3) slow gelation also opens the door to the inclusion of chemotactic agents following a specific spatial gradient, capable, for example, of triggering cell migration. Using a slow hydrolysis reaction to alter the pH, also allowed us to monitor the SAP aggregation process with light scattering methods, providing new insights into the gelation mechanism. We find that, in diluted and concentrated solutions gelation follows different pathways. This leads to gels with different microscopic dynamics and capability of trapping nanoparticles. Interestingly, well-controlled different gel morphologies within the scaffold, can also be used to achieve different kinetics of release of multiple drugs in vitro, where needed.

Further studies, combining the light scattering methods we used, with complementary techniques, such as time-resolved small-angle X-ray scattering [41], CD [46], and FTIR spectroscopy [48], are required, to shed full light on SAP gelation and to strengthen our findings.

The methodology we used in this work, can be extended to other base-triggered molecular gelators [19,20]. Understanding the possible effect of the urea–urease reaction in the self-assembly of urea-based SAPs [25], is particularly tempting. The fact that the gel we produced can be easily seeded with nanoparticles, is promising for possible application in drug delivery and tissue regeneration [8,64,65]. Particularly appealing, will be to study the dynamics of small objects, such as particles of different sizes [39,66], bacteria, viruses, and cells, embedded in weak gels, where microscopic motion is never completely arrested.

## 4. Materials and Methods

### 4.1. SAP Sample Preparation

In this investigation, we used the self-assembling peptide FAQRVPP−GGG−${\left(\mathrm{LDLK}\right)}_{3}{\mathrm{NH}}_{2}$ (Lot: A020/3, purity >99%) purchased from Nanomed3D Srl. This is a self-assembling peptide, making cross-beta structures. The peptides used in this work are derived from ${\mathrm{Ac}-\mathrm{LDLKLDLKLDLK}-\mathrm{CONH}}_{2}$, in the sense that they share the same self-assembling backbone, viz. ${(\mathrm{LDLK}}_{3})$. It has been fully characterized in the work of Gelain et al. [67]. SAPs were solid-phase microwave synthesized, by using the standard Fmoc approach. The molecular weight of the final product was evaluated through the MS technique. LC-MS spectra were recorded via a single quadrupole mass detector (Waters LC-MS Alliance 3100, Waters Corp., Milford, CT, USA), using a nebulizing nitrogen gas at 400l/min and a temperature of $250{\;}^{\circ }C$. The cone flow, capillary, and cone voltage, were respectively set at 40mL/min, 3.5kV, and 60V. HPLC purification of the synthesized peptide, was performed using a Waters binary HPLC on a Restek (Restek Corp., Bellefonte, PA, USA) preparative $C_{1}8$ column. The mobile phase consisted of a gradient of acetonitrile with 0.1% TFA and $H_{2}O$ with 0.1% TFA, over 25min. After HPLC purification and lyophilization, TFA salts were removed by dissolving the product (0.5%w/v) in 0.01M HCl solution, and lyophilized again.

The SAP ${\mathrm{FAQRVPP}-\mathrm{GGG}-(\mathrm{LDLK})}_{3}{\mathrm{NH}}_{2}$ we used, contains alternating charged hydrophilic and hydrophobic amino acid residues in its self-assembling backbone sequence, and is known to have a strong propensity to generate cross β-sheet structures. When the pH of the solution is sufficiently high, hydrophobic forces drive its assembly into cross-beta structures, yielding nanofibers featuring charged residues exposed to water, and hydrophobic ones buried in a hydrophobic inner pocket. Such molecular organization has been thoroughly described in the work of Gelain et al. [13].

Urea, urease (type III from Jack beans, U1500, Sigma Aldrich, St. Louis, MO, USA, 40,150U/g, Product Code: 1002597636) and acetic acid (Sigma Aldrich), were used without further purification.

The SAP stock solution, at 20g/L, was prepared by suspending the SAP powder in deionized water and sonicating for at least 20min, until a clear solution formed. The SAP stock solution was then filtered and kept in the fridge for a maximum of three days. Urease solutions were freshly prepared before use, dissolving the enzyme in distilled water and keeping it in the fridge for a maximum of 8h. In this way, we minimized the denaturation of the urease. We verified that the activity of the dissolved urease, if kept at $T=4{\;}^{\circ }C$, does not decrease appreciably in 10h. To prepare the gel, the SAP stock solution, diluted in an aqueous solution of acetic acid, was firstly added to the measured quantities of the urea solution, to tune the final pH of the gel. Then, the sample was gently mixed in an Eppendorf 1.5mL centrifuge tube. In a second phase, a controlled amount of a urease aqueous solution was added to the tubes using an electronic pipette. The whole solution was aspirated and dispersed back into the tube at least three times, in order to speed up and optimize the sample mixing. The entire mixing procedure lasted less than 30s. At the end, the sample was split in two parts: The first one was poured in the cuvette inside the PCI setup, the other half was used to fill the DLS cell. The filling of the cells in the two setups was synchronized, in order to ensure the same pH evolution and gelation kinetics in both the experiments and simplify their comparison.

As detailed in Section 2.3, we also studied the gelation in samples with PMMA particle tracers, having diameter σ=192nm (microparticles Gmbh) and stabilized with Pluronic^®^ F-127 (Sigma Aldrich). In this case, the previous sample preparation procedure was slightly modified, by initially diluting the SAP stock solution with an acetic acid solution seeded with the colloidal tracers.

### 4.2. Optical Methods: Dynamic Light Scattering and Photon Correlation Imaging

In a dynamic light scattering (DLS) experiment, the sample dynamics are probed on length scales of the order of the inverse of the scattering wave-vector q=(4πn/λ)sin(ϑ/2), where ϑ is the scattering angle, *n* is the refractive index of the solvent, and λ is the laser wavelength in a vacuum, by measuring the (normalized) time correlation function of the scattered intensity *I* (ICF):

(1) $$g_{2}\left(\tau ;t\right)=\frac{{\langle I(t)I(t+\tau )\rangle }_{\delta t}}{\langle I(t)\rangle {\langle I(t+\tau )\rangle }_{\delta t}},$$

where $\langle ...\rangle$ is experimentally a time average for a total duration δt, over the initial time *t* [68]. In all measurements we discuss, we have fixed $\vartheta =90^{\circ }$. When the dynamics of the studied process change with time, such as during a gelation process, where the value of scattered intensity and microscopic dynamics depend on time, δt has to be kept smaller than the timescale of the material’s time evolution.

When DLS is used to investigate arrested systems such as gels, it is important to recall some peculiar issues that characterize the ICF [69]. In fact, DLS is routinely used to study “fluid-like” materials, in which the scatterers are free to move in the solvent. With time, the system explores the full ensemble of possible configurations of the scatterer positions. The ICF previously defined, time-averaged over the duration of a single experiment, is equal to the ensemble average obtained repeating the experiment several times. If this condition is fulfilled, the material is known as *ergodic*. On the contrary, in gels and glasses, the scatterers move around fixed average positions. Due to this limited motion, these systems can only move in a limited region of the phase space and are known as *non-ergodic*. In this case, the time-averaged quantities measured on a particular sample, i.e., the ICF in a single DLS experiment, are temporal averages over only a subensemble of configurations. These averages do not correspond to the ones sampled over the whole of the phase space [70]. If we suppose that scattering volumes that are sufficiently large contain many uncorrelated regions, then the scattered electric field E(t), is a complex Gaussian variable, whose mean value is equal to zero. In ergodic, fluid-like systems, the intensity sampled by the detector will fluctuate in time, because the scatterer positions change in time. If the experimental time, δt, is sufficiently long, the light field explores all the possible Gaussian fluctuations and the time and ensemble averages are the same. Due to Gaussian statistical properties, for τ=0, $g_{2}\left(0,t\right)-1=1$, while for τ much longer than the characteristic time of the ICF, I(t) and I(t+τ) are uncorrelated, so that $g_{2}\left(\tau ,t\right)-1=0$. Thus, the contrast of the ICF, defined as $g_{2}\left(0,t\right)-g_{2}\left(\infty ,t\right)$, is maximum and equal to 1.

In the opposite case, of a sample that is completely arrested, the speckle pattern is rigid, because scatterers are frozen in a fixed configuration and the scattered light intensity measured by the detector is constant in time, so that $g_{2}\left(\tau ,t\right)-1=0$ for every delay time τ. We consider now the intermediate case, of a non-ergodic sample (like a gel or a glass), in which the motion of the scatterers is partially arrested. Here, the intensity pattern is characterized by both a fluctuating and non-fluctuating contribution. It is easy to demonstrate that, for τ=0 $g_{2}\left(0,t\right)-1<1$, because only a part of all of the possible intensity fluctuations is probed. Conversely, at large delay times, $g_{2}\left(\tau ,t\right)-1=0$, because the fluctuations become uncorrelated, as in the ergodic case. The reduction in the contrast in a DLS experiment, is therefore a hallmark of the formation of a (partially) arrested system.

Recovering the true ensemble averaged ICF in a typical DLS experiment, is a tedious and time consuming procedure, that requires the sample or the detector to be moved across a large number of different positions [70,71]. To overcome this limitation, especially annoying in the case of time-varying samples, multi-speckle light scattering techniques have emerged in the past 20 years. Among them, photon correlation imaging (PCI) provides, in a single measurement, the ICFs of the scattered light at distinct points within the sample [72,73]. The light scattered by the sample at a given angle ϑ, is collected by a stopped-down optical system, forming a speckled image on a multi-pixel sensor. In this work, we renounce to spatial resolution, averaging the sample dynamics over all the pixels of the camera. This choice allows us to obtain a fast spatial averaging of the ICF for samples characterized by very slow dynamics. In analogy with the ICF defined for DLS, the correlation index $c_{I}\left(\tau ;t\right)$, between two images taken at times *t* and t+τ, is introduced

(2) $$c_{I}\left(\tau ;t\right)=\frac{\langle I_{p}\left(t\right)I_{p}\left(t+\tau \right)\rangle }{\langle I_{p}\left(t\right)\rangle \langle I_{p}\left(t+\tau \right)\rangle }-1,$$

where $\langle \cdots \rangle$ is the spatial average over the whole image of the scattered intensity $I_{p}$, measured on each pixel. It is easy to show that $c_{I}\left(0;t\right)$, with the relative variance of the intensity in the image at time *t*. Usually, PCI is employed with samples with very slow dynamics, and the exposure time, $t_{e}$, of the camera, of the order of a few ms or less, is much shorter than the characteristic timescale of the speckle field evolution. In this case, $c_{I}\left(0;t\right)$ is constant during the experiment, and depends only on the setup configuration. To easily compare experiments obtained with different setups, it is useful to introduce the normalized degree of correlation, ${\hat{c}}_{I}\left(\tau ;t\right)=c_{I}\left(\tau ;t\right)/c_{I}\left(0;t\right)$. On the contrary, if the sample restructuring time is comparable with $t_{e}$ and evolves in time, the value of $c_{I}\left(0;t\right)$ also changes during the experiment. In particular, if the decay time of the ICF is longer than $t_{e}$, the speckle pattern is frozen, and different pixels measure very different signals. Conversely, when the exposure increases, the visibility of the speckle pattern progressively decreases. Over the duration of an exposure, each pixel integrates the light intensity, that in the meantime fluctuates. If $t_{e}$ is too long compared to the characteristic time of the sample restructuring, each pixel measures the same averaged value, and there is a limited spatial variation in the signal. Speckle-visibility spectroscopy [74] exploits this idea to characterize the sample dynamics. In our case, we monitor the variation in $c_{I}\left(0;t\right)$ for a fixed $t_{e}=10\;\mathrm{ms}$, during the gelation process, in order to have access to a timescale much faster than the delay time between two PCI images.

The detailed properties of our experimental setup can be found in [75,76]. We simply recall, that the scattering vector that we probed in our PCI and DLS setups is q≃23µm ${}^{-1}$.

### 4.3. pH Measurement

A Thermo Scientific^™^ ORION^™^ 9810BN microelectrode, linked to an ORION^™^ benchtop 420A pH/mV meter, was used to follow the pH evolution during the gelation process (system pH accuracy of ±0.005). The microelectrode, filled with a filling solution of KCl 4M and Ag/AgCl (ORION^™^ Cat. N. 900011), is characterized by a length of 120mm, and a ceramic junction and tip of 37×1.3mm. It could be used to investigate samples with a minimum volume of 500nL. The small footprint of the electrode allowed us to insert the electrode inside the optical cell of the PCI setup, probing a region around 2mm from the scattering volume. All measurements were conducted at room temperature. The microelectrode was stored according to the requirements of the producer and calibrated every three measures.

## Data Availability

The data underlying the results presented in this paper are not publicly available at this time, but may be obtained from the authors upon reasonable request.

## Abbreviations

The following abbreviations are used in this manuscript:

SAP	Self-assembling peptides
ECM	Extracellular matrix
GdL	Glucono-δ-lactone
CD	Circular dichroism
FTIR	Fourier-transform infrared spectroscopy
Fmoc	9-Fluorenylmethyloxycarbonyl
Tfa	Trifluoroacetate
HPLC	High-performance liquid chromatography
LC-MS	Liquid chromatography–mass spectroscopy
DLS	Dynamic light scattering
PCI	Photon correlation imaging
ICF	Intensity correlation function
